# Hypopharyngeal carcinoma in Finland from 2005 to 2014: outcome remains poor after major changes in treatment

**DOI:** 10.1007/s00405-022-07648-5

**Published:** 2022-09-12

**Authors:** Harri Keski-Säntti, Marjaana Luukkaa, Timo Carpén, Anna Jouppila-Mättö, Kaisa Lehtiö, Hanna Mäenpää, Kristiina Vuolukka, Tero Vahlberg, Antti Mäkitie

**Affiliations:** 1 Department of Otorhinolaryngology-Head and Neck Surgery, Helsinki University Hospital, University of Helsinki, P.O.Box 263, 00029 Helsinki, Finland; 2 Department of Oncology, Turku University Hospital and University of Turku, Turku, Finland; 3 Research Program in Systems Oncology, Faculty of Medicine, University of Helsinki, Helsinki, Finland; 4 Department of Pathology, Helsinki University Hospital and University of Helsinki, Helsinki, Finland; 5 Department of Otorhinolaryngology-Head and Neck Surgery, Kuopio University Hospital, Kuopio, Finland; 6 Department of Oncology, Oulu University Hospital and University of Oulu, Oulu, Finland; 7 Department of Oncology, Tampere University Hospital, Tampere, Finland; 8 Department of Oncology, Kuopio University Hospital, Kuopio, Finland; 9 Department of Clinical Medicine, Biostatistics, University of Turku, Turku, Finland

**Keywords:** Hypopharyngeal carcinoma, Hypopharyngeal SCC, Head and neck cancer

## Abstract

**Purpose:**

Hypopharyngeal carcinoma (HPC) is typically diagnosed at late stages, the patients tend to have serious co-morbidities, distant relapses are frequent, and the related mortality remains high. The treatment paradigm of HPC has remarkably changed from primary surgical approach toward definitive, platinum-based concomitant chemoradiotherapy (CRT). Our aim was to analyze the HPC treatment approaches and outcome in a nationwide series and to make a comparison with a previously published corresponding nationwide patient cohort from the period 1990–1999.

**Methods:**

We retrospectively reviewed all patients diagnosed with HPC at the five university hospitals in Finland between 2005 and 2014.

**Results:**

The cohort comprised 231 patients. Treatment with curative intent was offered for 175 (76%) patients and consisted of definitive radiotherapy (RT) or CRT in 156 (89%) patients, while 20 (11%) patients had primary surgery with or without adjuvant RT or CRT. The 5-year estimates for overall survival (OS) and disease specific survival (DSS) for the whole study group were 22.7% and 36.5%, respectively. For patients treated with curative intent, the 5-year estimates for OS and DSS were 29.4% and 44.3%, respectively.

**Conclusions:**

The treatment approach of HPC in Finland has changed thoroughly, as in the 1990s, 63% of HPC patients with curative treatment intent underwent primary surgery with or without RT, while in the current study, the primary treatment approach was non-surgical in 89% of the patients. However, the survival figures have not changed and remain dismal, but most of the few surviving patients now can retain their larynx.

## Introduction

Hypopharyngeal squamous cell carcinoma (HPC) is assumably the deadliest of all mucosal head and neck cancers. Not only is the cancer itself difficult to cure because of typically late diagnosis and frequent distant metastases, but also the HPC patients tend to be heavy users of alcohol and tobacco with significant comorbidities and often poor nutrition status. The reported survival figures for patients diagnosed with HPC have typically been between 13 and 38%, and with only modest improvement in recent years [1–5].

The treatment results of HPC in Finland (population 5.5 million) in the period 1990–1999 were reported by Laranne et al. in a nationwide series [6]. During that period, the mainstay of treatment was primary surgery and adjuvant radiotherapy (RT). Surgical treatment of HPC, however, in most cases is mutilating necessitating the removal of larynx. In the landmark studies in the 1990s, the larynx could be preserved in many patients with hypopharyngeal and laryngeal carcinoma without jeopardizing survival using induction chemotherapy followed by radiotherapy (RT) for responders [7, 8]. Thereafter, the justification of laryngectomy was questioned and the treatment paradigm of HPC was revolutionized toward organ sparing protocols. Approximately since the beginning of new millenium until these days, the preffered treatment for HPC in the Finnish national guidelines has been concomitant platinum-based chemoradiotherapy (CRT) with surgery as a salvage option for patients with treatment failures. As recommended in the internationally published guidelines [9], primary surgery is considered only rarely in patients with extensive tumors seriously affecting function and/or causing cartilage destruction.

Almost simultaneously with the change in the treatment paradigm, a novel technique for the delivery of RT was introduced. Intensity modulated radiation therapy (IMRT) is a computer-controlled high precision RT technique, which allows more meticulous target delineation compared to older techniques. IMRT has been used in the treatment of head and neck cancer in Finland since the first years of the new millenium. Later in the study period volumetric modulated arc therapy (VMAT) was also used. These modulated RT techniques (IMRT and VMAT) are now standard techniques in the treatment of this tumor group nationwide. Simultaneous integrated boost (SIB) allows simultaneous delivery of different doses to several different target levels. These techniques improve saving of normal structures. The positive effect of combining chemotherapy with RT in the treatment of head and neck cancer has been known for decades, cisplatin being the best documented single chemotherapeutic agent. Globally, different schedules of chemotherapy are being used. In Finland concomitant CRT with weekly low-dose cisplatin has been used for over two decades in the management of head and neck cancer. Administered weekly, cisplatin seems to be better tolerated and noninferior in efficacy compared to high dose cisplatin [10–12].

Since 1992, the Finnish Head and Neck Oncology Working Group has defined a national treatment protocol for head and neck cancer. The treatment results of different head and neck cancers are monitored by nationwide analyses on a regular basis, including the current study. In the yearly meetings of the Working Group, necessary updates are made in the treatment protocol. Now, we had special interest in investigating the impact of the above described major changes in the treatment of HPC. We aimed to retrospectively analyze the treatment results of all patients diagnosed with HPC in Finland between 2005 and 2014 and to compare the results with the previously published national data on HPC patients treated in the 1990s [6].

## Patients and methods

Institutional research permissions to conduct this retrospective nationwide study were obtained (Helsinki: §141, Nov, 9, 2018, Turku: T157/2018, Oulu: 196/2018, Kuopio: TJ 121/2018, Tampere: R18620). This research involved only patient charts, and therefore, no formal Research Ethics Board approval or informed consent was needed according to the Finnish legislation. Clinicopathological data of all patients who were diagnosed with previously untreated, biopsy proven squamous cell carcinoma of hypopharynx at the five university hospitals in Finland (population 5.5 million) during 10-year period 2005–2014 were reviewed. This study can be regarded as a population-based nationwide series as during the study period practically all HPC patients were treated at the five university hospitals. There may be some dropouts, however, as only since 2018 has the treatment of head and neck cancer been centralized to the five university hospitals by law. All patients were evaluated by a local multidisciplinary tumor board. The hospital records were reviewed and data on patient and tumor characteristics, treatment, and follow-up were recorded*.*

Primary surgical treatment was defined as upfront surgery including resection of the primary tumor. If only neck dissection was performed with consequent definitive oncological treatment of the primary tumor, the patient was categorized in the definitive RT or CRT group.

The treatment doses and techniques of RT were determined in each hospital resulting in slight variations across the centres. Individualized thermoplastic masks were used for immobilization and for accuracy of RT. The photon fields were verified with images during therapy according to local guidelines. Conventional three-dimensional (3D) conformal RT was used only among few patients mostly in the beginning of the study period. IMRT or VMAT were the most often used techniques, since these advanced computer techniques have been the preferred choice since 2005 in all centres. Simultaneous integrated boost (SIB) was used with IMRT in many centres. Radiotherapy was administered principally 5 days a week, and definitive treatment usually lasted for 7 weeks.

When treated with concurrent CRT, the standard radiation sensitizer used was intravenous cisplatin 40 mg/m^2^ weekly. During the study period when cisplatin was contraindicated, the treatment of choice could be monoclonal antibody such as cetuximab or another chemotherapeutics such as taxanes.

### Statistical analysis

Continuous variables are described as mean (standard deviation) or median (interquartile range) and categorical variables as number (percentage). The follow-up time was calculated from the date of diagnosis to the end of follow-up or the date of death. Survival curves for overall survival (OS) and disease-specific survival (DSS) were done with Kaplan–Meier method for all patients and for patients who were offered curatively intended treatment. Log-rank test was used to compare DSS curves by stage and treatment modality. *P* values of less than 0.05 were considered as statistically significant. Statistical analyses were carried out using the SAS System for Windows, release 9.4 (SAS Institute Inc., Cary, NC).

## Results

During the period 2005–2014, 231 patients were diagnosed with previously untreated HPC, of which 182 were males and 49 females. The mean age of the patients was 64.5 years (SD 9.9; range 34.8–91.6). The patient characteristics are demonstrated in Table 1.

**Table 1:** Patient and tumor characteristics

	*n* (%)
Gender	
Male	182 (79)
Female	49 (21)
Tumour subsite	
Pyriform sinus	64 (28)
Postcricoid	14 (6)
Aryepiglottic fold	13 (6)
Posterior wall	20 (9)
Overlapping sites	89 (39)
Not specified	31 (13)
Stage*	
I	4 (2)
II	11 (5)
III	42 (18)
IVA	142 (61)
IVB	21 (9)
IVC	11 (5)
Treatment intention	
Curative	176 (76)
Palliative	55 (24)
Treatment	
CRT	129 (56)
RT	26 (11)
SX ± (C)RT	20 (9)
Palliative	56 (24)
PEG tube	
Yes	190 (82)
No	41 (18)

*Sx* surgery, *CRT* chemoradiotherapy, *RT* radiotherapy

*UICC TNM Classification of Malignant Tumours, 7th edition

Smoking history was available for 205 patients, of which 188 (92%) were current or former smokers.

During the study period, 192 (83%) of the 231 patients died, of which 132 (69%) because of HPC and 60 (31%) for other reasons.

The mean and median follow-up time for the whole cohort were 2.69 years and 1.46 years, respectively (range 0.02–13.73). The mean and median follow-up time for the patients who were alive at the end of the study period (*n* = 39) were 6.28 years and 5.46 years, respectively (range 0.89–13.73).

Curatively intended treatment could be offered for 175 (76%) patients, while for 56 (24%) patients only supportive/palliative treatment was possible. Curatively intended treatment consisted of definitive RT in 26 patients (1 with prior neck dissection) and definitive CRT in 129 patients (4 with prior neck dissection). In 20 patients the primary treatment was surgery followed by RT in 7 and CRT in 12 patients. Primary surgery included 16 laryngectomies with partial or total pharyngectomy with or without neck dissection and in 4 patients endoscopic resection of the tumor with or without neck dissection.

In the definitive CRT group (129 patients), the mean treatment time was 50 days (median 49, range 41–84, *n* = 129), mean dose to tumor was 69.3 Gy (median 70, range 58–71, *n* = 129) and mean dose to ipsilateral metastatic lymph nodes was 68.6 Gy (median dose 70, range 49–70, *n* = 92). Neck was treated with an elective mean dose of 52.7 Gy (median 50, range 45–67, *n* = 127) and contralateral neck with an elective mean dose of 52.3 Gy (range 45–66, *n* = 119).

For the patients with definitive RT without concurrent chemotherapy (*n* = 26), the mean treatment time was 50 days (median 49, range 43–66, *n* = 26), and the mean dose to tumor 68 Gy (median 70, range 54–71, *n* = 26). The mean dose to ipsilateral metastatic lymph nodes was 66.6 Gy (median 68.5, range 54–70), mean ipsilateral elective neck dose 52 Gy and contralateral neck was treated with an elective mean dose of 51.9 Gy.

In the patients treated with primary surgery followed by adjuvant RT or CRT (*n* = 10) the mean treatment time of RT after surgery was 43.2 days (median 43.5, range 38–54). Mean dose to tumor was 62.5 Gy (median 63, range 58–66), mean dose to ipsilateral neck metastasis was 62.4 Gy (median 63, range 59–66), mean dose to contralateral metastasis was 60 Gy and to contralateral elective neck 51.9 Gy. For 10 patients the treatment was surgery only.

Altogether 110 (87%) out of the 129 patients with curatively intended CRT had cisplatin as a radiation sensitizer. Four patients had cetuximab and for three patients cisplatin was changed either to paclitaxel, carboplatin or cetuximab. One patient had two cycles of carboplatin and etoposide and another patient had one cycle of cisplatin and fluorouracil. Among 10 patients the radiosensitizer is not known retrospectively.

Six to eight cycles of chemotherapy was administered to 78 (62.4%) patients among those with treatment with curative intent, 34 (27.2%) had four to five cycles, and 13 (10,3%) had one to three cycles. In four patients the number of cycles was unknown.

Fifteen patients received palliative RT and the mean dose was 41.4 Gy (median 45.9, range 8–63).

Fifty-three (34%) out of 155 patients treated with definitive RT or CRT were assessed to have locoregional residual disease after treatment. Thirty (57%) of these patients were operated: 10 had laryngopharyngectomy, and 20 patients neck dissection only. Of these 30 operated patients, 4 (13%) were alive in the end of the follow-up.

Of the 123 patients initially scheduled for curatively intended treatment and with complete response achieved, 67 (54%) experienced recurrent disease in the follow-up. Locally and/or regionally recurrent disease was detected in 30 (24%) patients (17 local, 22 regional) and distant metastasis in 45 (37%) patients. Sixty-three (94%) of these 67 patients with recurrent disease died during the study period.

**Fig. 1:**
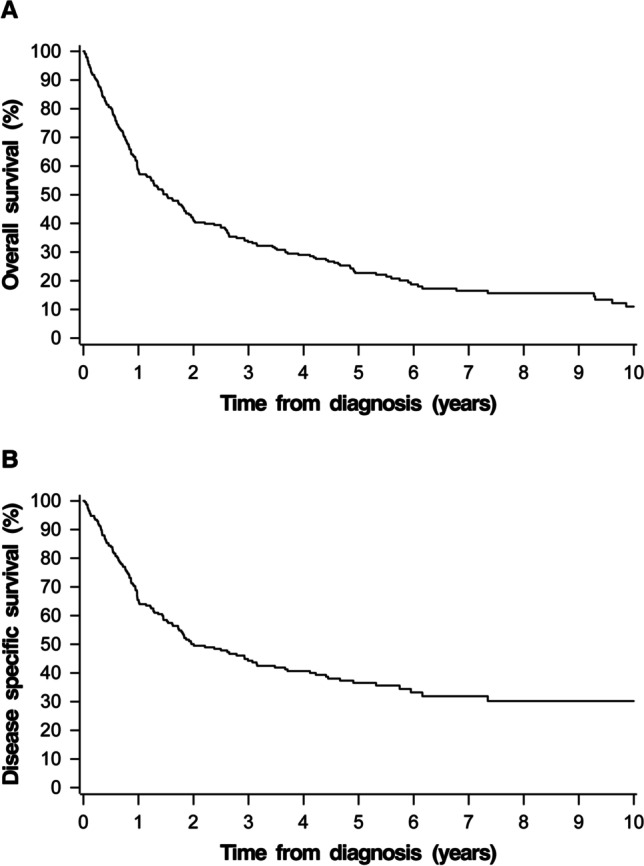
Kaplan–Meier survival curve for overall survival (**A**) and disease specific survival (**B**) for the whole study group

**Fig. 2:**
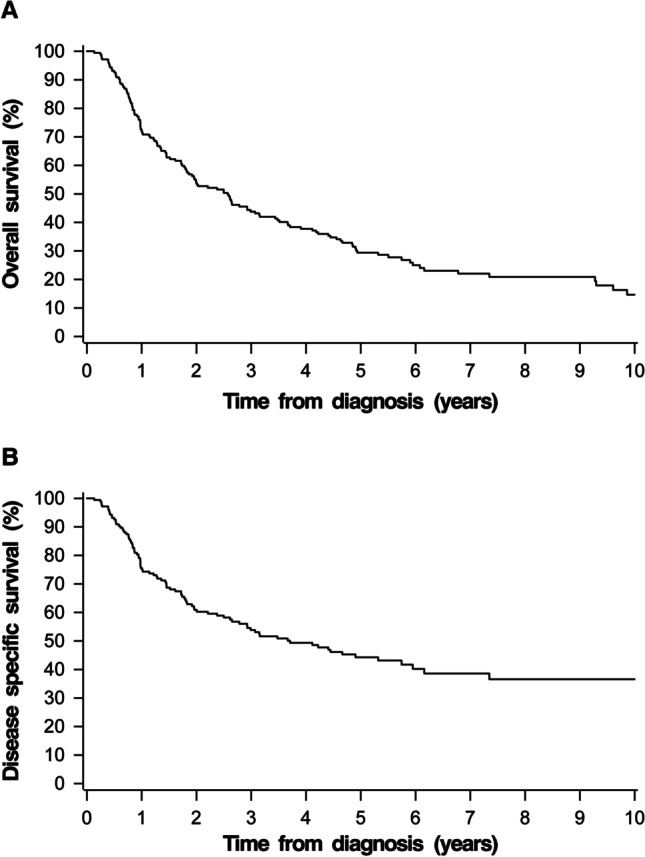
Kaplan–Meier survival curve for overall survival (**A**) and disease specific survival (**B**) for the patients who were offered curatively intended treatment

**Fig. 3:**
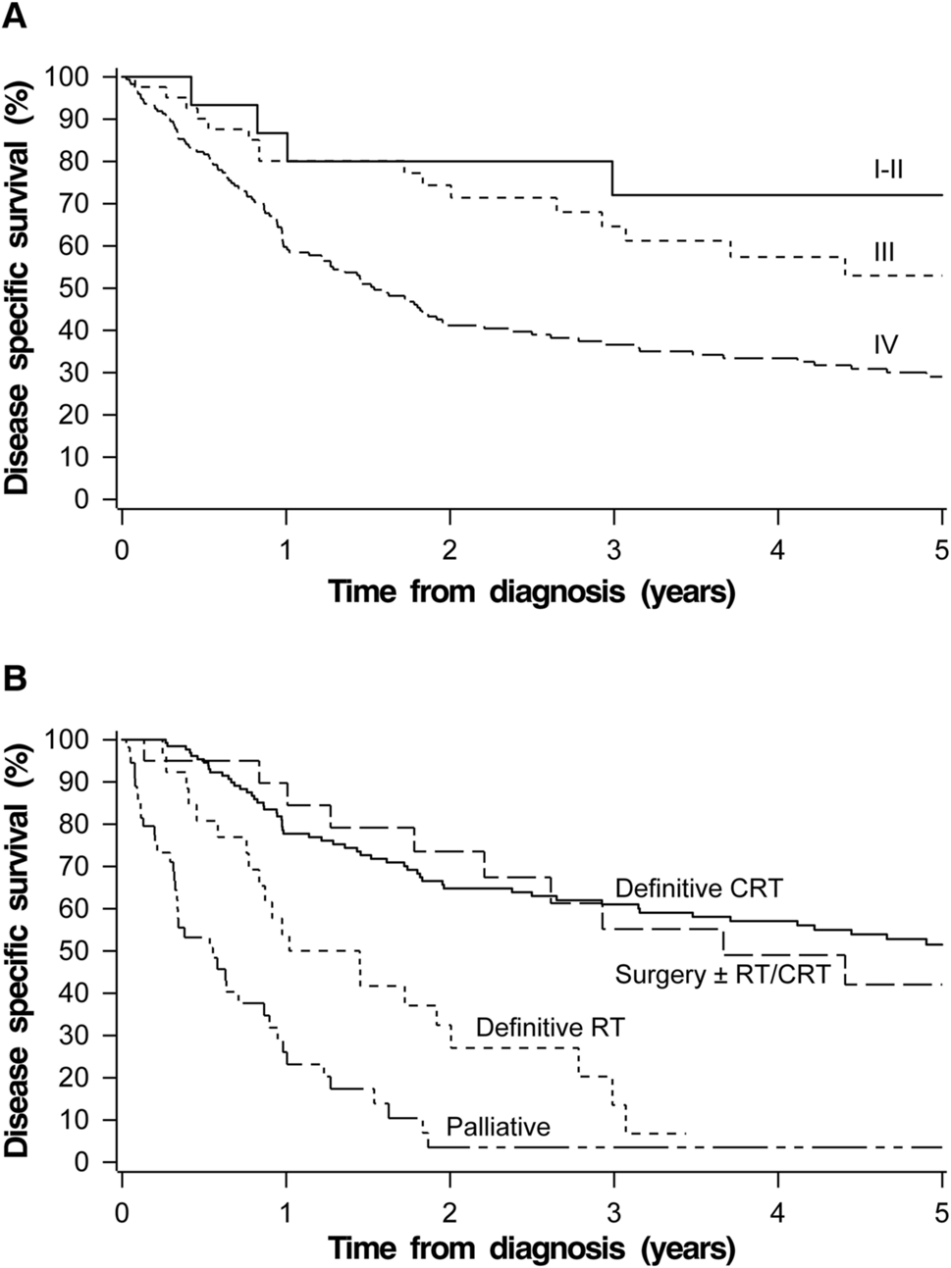
Kaplan–Meier survival curve for disease specific survival by stage (**A**) and treatment modality (**B**) for the whole study group. Differences were significant between stages (overall *p* < 0.001); significant pairwise differences between I and II vs. IV (*p* < 0.001) and III vs. IV (*p* < 0.001). Differences were significant between treatment modalities (overall *p* < 0.001); all pairwise comparisons were significant (*p* < 0.001 for all other comparisons, except *p* = 0.013 for definite RT vs. surgery ± RT/CRT)

The 5-year and 10-year Kaplan–Meier estimates for overall survival (OS) and disease specific survival (DSS) for the whole cohort were 22.7% and 11.0%, and 36.5% and 30.2%, respectively (Fig. 1). For the patients with curatively intended treatment, the 5-year and 10-year Kaplan–Meier estimates for OS and DSS were 29.4% and 14.6%, and 44.3%, and 36.3%, respectively (Fig. 2). The 5-year DSS was 72.0% for stage I–II, 52.9% for stage III, and 29.0% for stage IV disease. The DSS by stage and treatment modality is demonstrated in Fig. 3.

The median OS time for patients with palliative/supportive treatment only was 0.34 years (range 0.02–5.59).

The p16 status (a surrogate marker for HPV infection) of the tumor was known in 53 patients, of which 13 (25%) were p16 positive. Nine (69%) of the patients with p16 positive and nine (23%) of the patients with a p16 negative tumor were alive in the end of the follow-up.

## Discussion

We investigated a nationwide series of HPC patients over a 10-year period comprising 231 patients treated between 2005 and 2014 at the five university hospitals in Finland. Major changes in the treatment paradigm of this notorious disease have taken place since the 1990s, and the present results were compared with an earlier 10-year report by Laranne et al. covering the years 1990–1999 in Finland [6]. In that study, 89% of the patients had stage III–IV disease at the time of diagnosis, corresponding to 93% in the current study indicating no improvement at all in terms of earlier diagnostics. Consequently, the proportion of patients to whom curatively intended treatment could be offered remains practically unchanged: 79% in the 1990s series vs. 76% in our data. The preferred treatment approach, instead, has totally changed since the 1990s. Of the patients with curative treatment intent in the 1990s series, 63% had primary surgery with or without postoperative RT, while 28% were treated by definitive RT, and 10% by definitive CRT. In the current series, the corresponding figures for primary surgery, RT, and CRT were 11%, 15%, and 74%, respectively. Thus, up to 89% of the patients in the current series with curative treatment intent, were primarily offered non-surgical treatment. A comparison of the current data with the 1990s series is presented in Table 2.

**Table 2:** Current HPC data compared to earlier series from the 1990s [6]

	1990–1999	2005–2014
	*n* (%)	*n* (%)
Sex		
Male	111 (74)	182 (79)
Female	38 (26)	49 (21)
Stage		
I	6 (4)	4 (2)
II	11 (7)	11 (5)
III	32 (22)	42 (18)
IV	100 (67)	174 (75)
Treatment		
Sx ± RT/CRT	74 (50)	20 (9)
RT	33 (22)	26 (11)
CRT	12 (8)	129 (56)
Palliative	31 (21)	56 (24)
Survival all	%	%
5-year OS	21	23
5-yearDSS	22	37
Survival curative intent		
5-year DSS	27	44

*HPC* hypopharyngeal carcinoma, *Sx* surgery, *RT* radiotherapy, *CRT* chemoradiotherapy, *OS* overall survival, *DSS* disease specific survival

Like in our 1990s series, and in the study by Burbure et al. [5], RT alone was associated with worse survival compared with CRT and primary surgical treatment. Conclusions from this, however, can only be made with extreme caution, because in a retrospective study setting, the selection of patients in different treatment groups is highly biased. For example, the most probable reasons for omitting chemotherapy are high age and remarkable comorbidities.

The 5-year estimates for OS and DSS for the whole study group in the present study were 23% and 37%, respectively. In the 1990s patient cohort the corresponding figures were 21% and 22%, respectively. In an even older study consisting of 162 HPC patients treated at the Helsinki University Hospital between 1958 and 1982, the estimate for 5-year OS was reported 11% for all patients and 18% for patients treated with curative intent [13]. Thus, we can demonstrate only a very slight improvement in survival figures of HPC patients in Finland over a 50-year period. This finding is consistent with a recent report by Koskinen et al. [14]. The major benefit from the changes in the treatment toward organ sparing protocols seems to be the preservation of larynx of most of the few surviving patients. Of the 39 patients alive at last follow-up in the current series, only 3 had had laryngopharyngectomy performed. Four (10%) out of these surviving patients did not have PEG tube removed (one with laryngopharyngectomy) indicating that swallowing function was reasonably well-preserved in the majority of the patients after CRT.

Our results are fairly well in line with other recent reports. In a Danish study by Jakobsen et al. [2] the 5-year OS figures of HPC patients increased from 13.4% in the period 1980–1985 to 26.9% in the period 2010–2014. In another recent report by Petersen et al., as in our study, a remarkable shift toward organ preservation therapies was demonstrated in the Netherlands since the 1990s [4]. In that study, only slight improvement in 5-year OS figure was found: from 28% in period 1991–2000 to 34% in period 2001–2010. We have previously reported the treatment results of HPC patients treated by definitive RT or CRT using IMRT at the Helsinki University Hospital between 2002 and 2010 [15]. In this series consisting of 45 patients all with curative treatment intent, the 5-year estimates for OS, DSS and locoregional control were 31%, 45%, and 64%, respectively. All surviving patients in this study group could retain functioning larynx. A part of this study cohort was included also in the current study.

Hypopharyngeal carcinoma is known for its propensity to metastasize distantly. This was shown in a recent review article on risk factors for distant metastasis in head and neck cancer, in which hypopharyngeal primary site significantly increased the risk [16]. In the current series, 45 (26%) of the 176 patients receiving curatively intended treatment developed distant metastasis in the follow-up. In our previous study consisting of 45 HPC patients with curatively intended treatment by RT or CRT, 14 (31%) were later diagnosed with distant metastasis [15].

In oropharyngeal cancer, Human Papillomavirus (HPV) is known to play a significant role in carcinogenesis. In oropharyngeal cancer, HPV positivity is known to be a strong positive prognostic sign. In HPC the role of HPV is less clear. In the study by Marshall et al., 167 (26%) of the 640 HPC patients with HPV status available, had HPV-positive tumors, which correlated significantly with improved OS and DSS compared to patients with HPV-negative tumors [17]. Correspondingly, in our study group there were 53 patients with p16 status available, and 13 (25%) of them were p16 positive (a surrogate marker for HPV infection). The number of these patients is too limited for any strict conclusions. It is, however, interesting to note, that nine (69%) of the 13 patients with a p16-positive tumor were alive in the end of the follow-up, while only nine (23%) of the 40 patients with a p16-negative tumor survived the study period.

Some recent papers call for reappraisal of the current nonsurgical treatment paradigm of HPC and suggest, that primary surgical treatment should probably be considered more often at least in selected HPC patients to optimize survival. In a retrospective study by Tsai et al., primary surgical treatment and definitive CRT were compared in a series of 652 patients with stage III–IV HPC [3]. Interestingly, surgically treated patients with stage IVA disease had significantly better OS and disease-free survival figures, while no difference in survival between treatment groups was found in patients with stage III and IVB disease. In the study by Petersen et al., survival of HPC patients with a T4 tumor was found significantly better after surgery and adjuvant RT compared to patients with definitive RT or CRT [4]. According to the authors, the assumed equivalence of organ preservation and laryngectomy may require reconsideration for T4 disease. Likewise, in the study by Hochfelder et al., consisting of 6055 subjects with stage III–IV HPC, there was a survival benefit for surgically treated patients; the difference in survival was not striking but appeared constant [1]. In some centres, transoral approaches are routinely used for larynx sparing surgical treatment of HPC. These approaches were reviewed in a meta-analysis by De Virgilio et al., which included 10 articles on transoral laser surgery and five on transoral robotic surgery [18]. The combined OS at 36 months of follow-up was 66.4% and the laryngeal function preservation cumulative rate was 94%. It must be noted, however, that patients in these studies are highly selected with many lower stage cases included. In the lack of prospective, randomized trials comparing different treatment approaches, the optimal treatment of HPC remains obscure.

In conclusion, since the 1990s, the preferred treatment modality for HPC in Finland has thoroughly changed from primary surgery toward definitive oncological treatment: in the present series of 231 HPC patients treated between 2005 and 2014, 89% of patients for whom curatively intended treatment could be offered were treated by definitive RT or CRT. However, the survival figures remain practically unchanged and dismal during the past decades. Hypopharyngeal carcinoma still is a disease diagnosed at late stages in patients with serious co-morbidities and with poor prognosis. The major improvement for the few surviving patients is the laryngeal preservation.
